# Mission Hospitals and Publicity

**Published:** 1918-02-09

**Authors:** J. Aikman Wilson

**Affiliations:** Asst. Sec., C.M.S. Church Missionary Society, Salisbury Square, London, E.C. 4


*To the Editor of* The Hospital.

### Mission Hospitals and Publicity.

*To the Editor of* The Hospital.

Sir,—Our attention has been drawn to a paragraph which appeared in The Hospital of date January 19, 1918, headed "Gaza Hospital." The last two sentences of the paragraph in question read as if (1) the C.M.S. did not supply information as to its work to the press, or (2) is unwilling to supply such information when asked for it.

In light of the fact that you applied to us for information, and that we immediately lent you photographs and gave you all the latest news we had received on December 3, 1917, we submit that your phrase "such meagre information as is usually available" does not give a fair impression.

If, however, you wish us to keep you posted with regard to any important developments in our medical missionary work, I have no doubt that this can be arranged. Please let us know.—Yours faithfully,
J. Aikman Wilson, Asst. Sec., C.M.S.

Church Missionary Society, Salisbury Square,
London, E.C. 4, January 25, 1918.

[We deal with this letter in a Note this week.—Ed. The Hospital.]

